# Correction: Dye-sensitized electron transfer from TiO_2_ to oxidized triphenylamines that follows first-order kinetics

**DOI:** 10.1039/c7sc90080e

**Published:** 2018-01-26

**Authors:** Brian N. DiMarco, Ludovic Troian-Gautier, Renato N. Sampaio, Gerald J. Meyer

**Affiliations:** a Department of Chemistry , University of North Carolina at Chapel Hill , Chapel Hill , North Carolina 27599-3290 , USA . Email: gjmeyer@email.unc.edu

## Abstract

Correction for ‘Dye-sensitized electron transfer from TiO_2_ to oxidized triphenylamines that follows first-order kinetics’ by Brian N. DiMarco *et al.*, *Chem. Sci.*, 2018, DOI: 10.1039/c7sc03839a.



## 

The authors regret that Scheme 1 is incorrect in the original manuscript as an arrow was not properly displayed. The correct scheme is shown below.

**Scheme 1:**
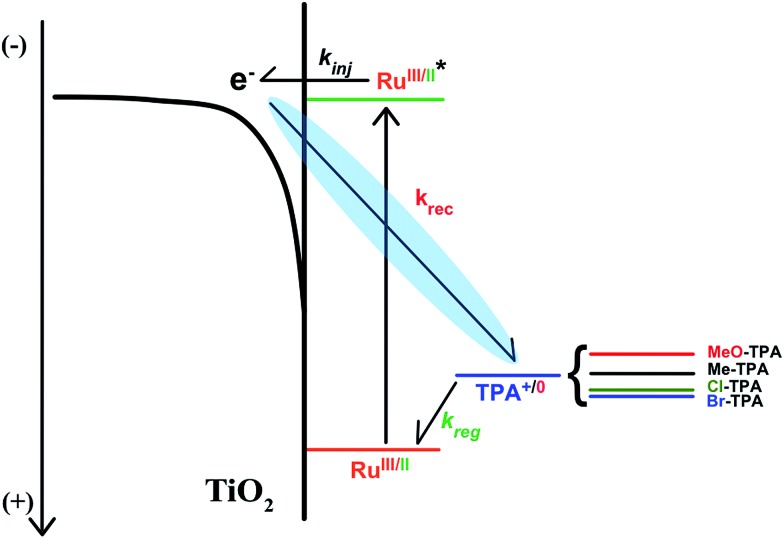
Mechanism for the photoinitiation of the desired reaction. Visible light absorption by the ruthenium sensitizer induced rapid excited-state electron injection to the acceptor state of TiO_2_, *k*_inj_ > 10^8^ s^–1^. The oxidized sensitizer is then regenerated by triphenylamine (TPA) with a rate constant *k*_reg_. This sequence provides the reactants for the desired charge recombination reaction of the injected electron with the oxidized triphenylamine redox mediator (*k*_rec_) that was quantified over a 0.5 eV change in driving force.

The Royal Society of Chemistry apologises for these errors and any consequent inconvenience to authors and readers.

